# Study on the Corrosion Behavior of Additively Manufactured NiCoCrFe_y_Mo_x_ High-Entropy Alloys in Chloride Environments

**DOI:** 10.3390/ma18194544

**Published:** 2025-09-30

**Authors:** Chaoqun Xie, Yaqing Hou, Youpeng Song, Zhishan Mi, Fafa Li, Wei Guo, Dupeng He

**Affiliations:** 1 Faculty of Materials Science and Engineering, Jiangsu University of Science and Technology, Zhenjiang 212100, China; xiechaoqun77@163.com; 2 Research Institute of Advanced Materials, Shenzhen 518048, China; 3 Faculty of Materials Science and Engineering, University of Science and Technology Beijing, Beijing 100081, China

**Keywords:** high-entropy alloy, FeCoCrNiMo, laser powder bed fusion, corrosion resistance, passive film

## Abstract

This study aims to determine the optimal Mo content for corrosion resistance in two alloys, FeCoCrNiMo_x_ and Fe_0.5_CoCrNiMo_x_. The alloys were fabricated using laser powder bed fusion (LPBF) technology with varying Mo contents (x = 0, 0.05, 0.1, 0.15). The corrosion behavior of these alloys was investigated in 3.5 wt.% NaCl solution at room temperature and 60 °C using electrochemical testing and X-ray photoelectron spectroscopy (XPS). The results show that all alloys exhibit good corrosion resistance at room temperature. However, at 60 °C, both alloys without Mo addition exhibit severe corrosion, while the Fe_0.5_CoCrNiMo_0.1_ alloy demonstrates the best corrosion resistance while maintaining the highest strength. The enhanced corrosion resistance is attributed to the optimal molybdenum addition, which refines the passive film structure and promotes the formation of Cr_2_O_3_. Furthermore, molybdenum oxide exists as MoO_4_^2−^ ions on the surface of the passive film, significantly improving the alloy’s corrosion resistance in chloride-containing environments.

## 1. Introduction

Corrosion is one of the three primary degradation modes of engineering materials [1], causing substantial economic losses in critical sectors such as aerospace, energy, chemical engineering, and marine engineering [2]. Global assessments indicate that corrosion-related costs amount to approximately 3.34% of gross domestic product (GDP), while the associated safety hazards and maintenance demands are increasingly emphasized [3]. With the expansion of human activities into aerospace and deep-sea environments, alloys are expected to withstand more extreme corrosive conditions, demanding superior mechanical performance and corrosion resistance [4]. Consequently, the design and development of novel corrosion-resistant alloys represent both an economic imperative and a technological frontier [5].

High-entropy alloys (HEAs), composed of five or more elements in near-equiatomic proportions, provide a disruptive alternative to conventional alloy design dominated by one or two principal elements [6,7]. Since their inception by Yeh [6] and Cantor [8] in 2004, HEAs have garnered significant interest owing to four core effects—high entropy, lattice distortion, sluggish diffusion [9], and the “cocktail effect.” These unique mechanisms endow HEAs with exceptional stability, mechanical strength, high temperature stability, and corrosion resistance, positioning them as promising candidates for next-generation structural applications [7,10,11].

Among HEA families, FeCoCrNi-based systems are particularly attractive due to their simple composition, stable FCC structure, and balanced mechanical properties across a wide temperature range [7,8,12,13,14]. Composition optimization, such as Fe reduction (e.g., Fe_0_._5_CoCrNi), has been shown to enhance the strength–ductility synergy [15]. Furthermore, minor alloying additions can significantly alter both mechanical and corrosion properties without destabilizing the solid-solution phase [16,17,18,19,20,21,22]. For example, Mo improves pitting resistance [23,24,25,26,27,28], Ti enhances both strength and corrosion resistance [29], Al promotes FCC-to-BCC transformation while stabilizing passive films [28,30], and Nb contributes to pitting resistance by a mechanism similar to Mo [31]. These insights highlight the critical role of microalloying in tuning the passivation behavior of FeCoCrNi-based HEAs.

Laser powder bed fusion (L-PBF), which is alternatively called Selective laser melting (SLM), offers a powerful means of fabricating HEAs with tailored microstructures [32]. Its extremely high cooling rate (>10^5^ K/s) results in refined grains [33,34,35], while repetitive melting–solidification cycles during layer-by-layer deposition introduce an intrinsic heat treatment (IHT) effect [36]. These features often yield distinct microstructural and functional characteristics compared with conventionally processed HEAs, presenting unique opportunities for performance optimization.

This study systematically investigates the role of Mo additions in FeCoCrNi and Fe_0.5_CoCrNi alloys fabricated via L-PBF. Alloys with varying Mo contents (x = 0, 0.05, 0.1, 0.15) were evaluated in terms of microstructure, mechanical performance, and electrochemical behavior in 3.5 wt.% NaCl solution at room temperature and 60 °C. The results demonstrate that while all alloys exhibit good corrosion resistance at room temperature, Mo-free alloys suffer severe degradation at elevated temperature. In contrast, Fe_0.5_CoCrNiMo_0.1_ combines the highest strength with superior corrosion resistance, owing to Mo-induced optimization of the passive film structure and enhanced Cr_2_O_3_ formation. These findings provide fundamental insights into Mo-doping mechanisms and establish design guidelines for engineering FeCoCrNi-based HEAs in aggressive environments.

## 2. Materials and Methods

### 2.1. Experimental Material

In our experiment, the raw materials consisted of Fe, Co, Cr, Ni, and Mo powders of 99.99% purity, characterized by particle sizes in the range of 15–53 μm and a spherical morphology of good quality (Jiangsu Vilory Advanced Materials Co., Ltd., Xuzhou, China). The powders exhibited good sphericity and flowability. To ensure precise compositional control, each elemental powder was weighed using an electronic balance (CN-LQC50002, Kunshan, China) with an accuracy of 0.001 g, and the weighing error was controlled within ±0.002 g. Compared with pre-alloyed powders, the direct use of elemental powders provides greater flexibility in alloy design while significantly reducing both the time and cost associated with powder preparation.

The theoretical compositions of the designed CoCrNiFe_y_Mo_x_ alloys are listed in Table 1. The weighed powders were mixed in a ball mill (YG-L, Jining, Shandong, China) for 5 h to achieve homogeneous distribution. The CoCrNiFe_y_Mo_x_ (y = 0,0.5; x = 0, 0.05, 0.1, 0.15) alloys were fabricated by laser powder bed fusion (L-PBF) using a DLM-120HT additive manufacturing system (Hangzhou Dedi Intelligent Manufacturing Co., Ltd., Hangzhou, China) under a high-purity argon atmosphere. For convenience, these alloys are denoted as Mo_0_, Mo_0.05_, Mo_0.1_, Mo_0.15_, Fe_0.5_Mo_0_, Fe_0.5_Mo_0.05_, Fe_0.5_Mo_0.1_, and Fe_0.5_Mo_0.15_, respectively, throughout the text.

During fabrication, the processing parameters were fixed as follows: layer thickness of 25 μm, hatch spacing of 80 μm, laser spot diameter of 100 μm, and a rotation angle of 67° between successive layers to minimize residual stress during printing [37,38]. The laser power was set to 200 W, 220 W, and 240 W, and the corresponding scanning speeds at each power level were 500 mm/s, 600 mm/s, and 700 mm/s. Each sample was subjected to three re-melting cycles to ensure complete melting and uniform elemental distribution. For each composition, the sample with the highest density and best surface quality among the nine processing conditions was selected for electrochemical corrosion testing, thereby minimizing the influence of surface defects on corrosion behavior.

**Figure 1:**
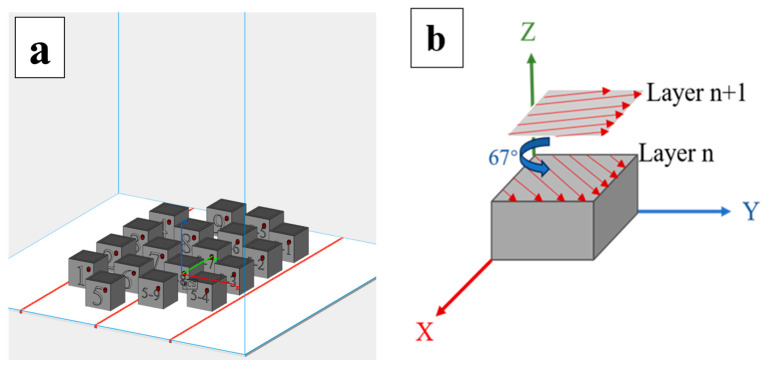
(**a**) Schematic diagram of sample preparation and (**b**) laser scanning strategy.

Representative fabricated samples of Mo_0_ and Fe_0.5_Mo_0_, together with the scanning strategy, are illustrated in Figure 1. The as-built specimens were detached from the substrate using electrical discharge wire cutting and subsequently sectioned into specimens of 10 mm× 10 mm× 3 mm to meet the dimensional requirements of subsequent tests.

### 2.2. Microstructural Characterization

The phase structures of FeCoCrNiMo_x_ and Fe_0.5_CoCrNiMo_x_ alloys were analyzed using X-ray diffraction (XRD, Rigaku DMAX-2600, Tokyo, Japan) with Cu Kα radiation, operated at 40 kV and 40 mA, over a 2θ range of 10–90° at a scanning rate of 8°/min. The microstructural morphology and elemental distribution were characterized using a scanning electron microscope (SEM, Apreo 2S, Thermo Scientific, Waltham, MA, USA) equipped with an energy-dispersive spectroscopy (EDS, Apreo 2S, Thermo Scientific, Waltham, MA, USA) detector. Prior to SEM observation, the prepared samples were sequentially ground with SiC papers of 320, 600, 800, 1200, and 1500 grit. For subsequent passive film analysis by XPS, the surfaces were further polished to 1 μm. After grinding and polishing, specimens were cleaned in ethanol and air-dried. For metallographic etching, aqua regia (HCl:HNO_3_ = 3:1) was used, and samples were etched for 20 s at room temperature.

### 2.3. Hardness Tests

Hardness tests were conducted using a Vickers hardness tester (MH-500, HENGYI PRECISION INSTRUMENT Co., Ltd, Shanghai, China). Prior to testing, the specimens were ground and polished mechanically to obtain a smooth finish. A load of 500 gf was applied during testing, with a dwell time of 15 s. For each specimen, five different regions were selected, and five indentations were measured per region. The average hardness value was then calculated.

### 2.4. Electrochemical Testing

Electrochemical measurements were conducted using a CS310M workstation (WUHAN CORRTEST INSTRUMENTS CORP., Ltd, Wuhan, China). The FeCoCrNiMo_x_ and Fe_0.5_CoCrNiMo_x_ alloys were employed as working electrodes, with a saturated calomel electrode (SCE) serving as the reference and a platinum sheet as the counter electrode. The electrolyte was a 3.5 wt.% NaCl solution at both room temperature and 60 °C. To avoid instability of the SCE potential, the electrode was not immersed for extended periods above 70 °C.

Prior to the experiment, the open-circuit potential was monitored for 3600 s to ensure its stabilization. Potentiodynamic polarization tests were then carried out from –0.5 V_SCE_ to +1.5 V_SCE_ at a scan rate of 0.5 mV/s. Electrochemical impedance spectroscopy (EIS) was conducted by applying an AC perturbation at 0 V_SCE_ to maintain passive film stability. The frequency range was 100 kHz–0.01Hz with an amplitude of 10 mV. Equivalent circuit models were constructed, and impedance data were fitted using ZSimpWin software (Version 3.30).

### 2.5. XPS Analysis of Passive Films

A stable passive film was ours, following OCP stabilization. The passive films on Mo_0_ and Mo_0.1_ alloys were examined formed on the specimen surface by polarizing the samples in a NaCl solution at 0 V_SCE_ for 3 h using X-ray photoelectron spectroscopy (XPS, Thermo Scientific K-Alpha, Waltham, MA, USA). Argon ions sputtering was used to remove surface contamination prior to analysis. XPS spectra were processed with Avantage software (Version 5.9), calibrating all binding energies to the C1s peak at 284.8 eV.

## 3. Results

### 3.1. Phase Constitution, Microstructure, and Properties

#### 3.1.1. Calculation of Solid-Phase Molar Fractions During Non-Equilibrium Solidification

**Figure 2:**
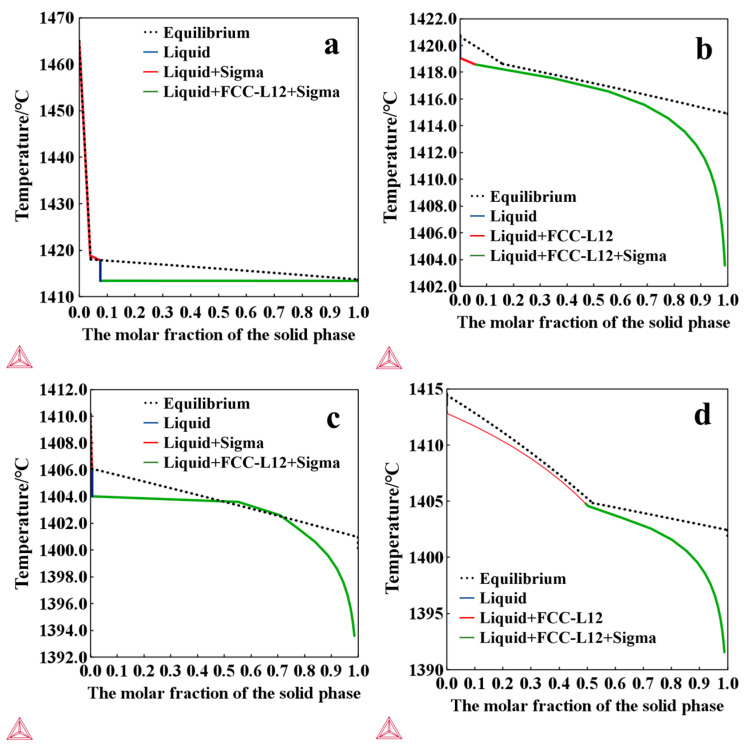
Molar fraction of the solid phase during non-equilibrium solidification (**a**) Mo_0.2_, (**b**) Mo_0.15_, (**c**) Fe_0.5_Mo_0.2_, (**d**) Fe_0.5_Mo_0.15_.

Excessive addition of Mo is known to promote the formation of the σ phase (a Cr- and Mo-rich intermetallic) [39], which can induce local corrosion in adjacent Cr and Mo-depleted regions [23]. To avoid σ-phase precipitation, the Mo content in this study was carefully controlled to ensure retention of a single-phase FCC structure. Prior to experimentation, the non-equilibrium solidification behavior of FeCoCrNiMo high-entropy alloys was thermodynamically simulated using Thermo-Calc 2023a. In 2021, Thermo-Calc released a solute trapping model tailored for the ultra-rapid solidification process in additive manufacturing. In the present study, the non-equilibrium Scheil solidification module incorporating the solute trapping model was employed, with a scanning speed of 500 mm/s. As shown in Figure 2, when the alloy composition was Fe:Co:Cr:Ni:Mo = 1:1:1:1:0.2, the system exhibited a strong tendency for σ-phase precipitation during solidification. In contrast, reducing the Mo atomic fraction to Fe:Co:Cr:Ni:Mo = 1:1:1:1:0.15 favored the preferential formation of a Face-centered Cubic (FCC) phase, thereby effectively suppressing σ-phase formation, mitigating the risk of localized corrosion, and ultimately determining the maximum Mo content adopted in the investigated alloy systems.

#### 3.1.2. XRD Analysis

**Figure 3:**
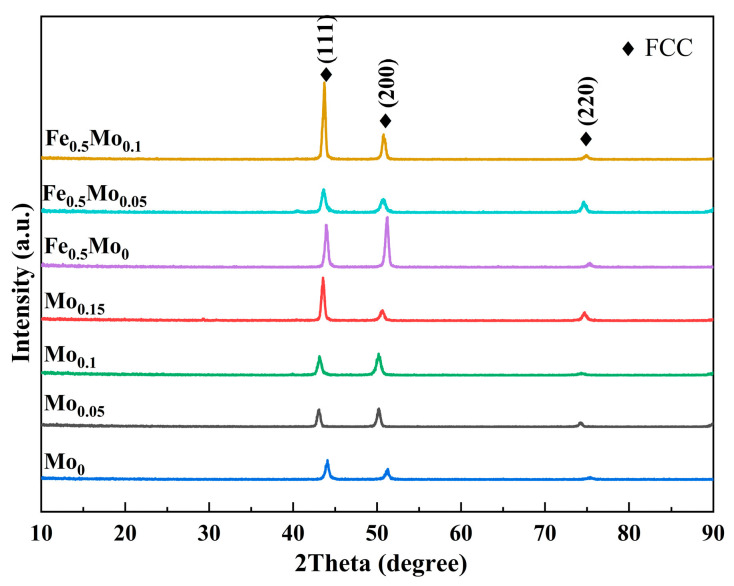
XRD spectra of HEAs with different Mo contents.

Figure 3 presents the XRD test results of the FeCoCrNiMo_x_ and Fe_0.5_CoCrNiMo_x_ HEAs fabricated by L-PBF. In conjunction with the calculated results shown in Figure 2, all samples with varying Mo contents exhibit a single-phase FCC structure, which can be primarily attributed to the unique high-entropy effect inherent to HEAs. With increasing Mo content, the diffraction peaks progressively shift toward lower angles, mainly due to the incorporation of Mo atoms with relatively larger atomic radii, which increases the lattice parameter and consequently enlarges the interplanar spacing. Notably, no additional diffraction peaks are detected, indicating that no considerable formation of secondary phases occurred during the L-PBF process. Nevertheless, given that XRD is unable to detect phases with a content below 3–5%, the presence of trace amounts of the σ phase cannot be completely excluded.

#### 3.1.3. Microstructural Analysis

**Figure 4:**
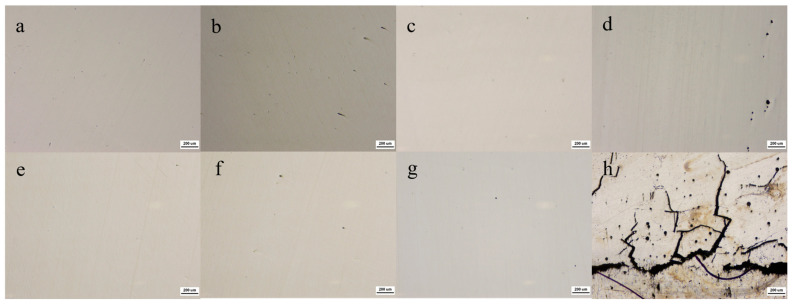
Printed XZ plane optical microscopic image (uncorroded) (**a**) Mo_0_, (**b**) Mo_0.05_, (**c**) Mo_0.1_, (**d**) Mo_0.15_, (**e**) Fe_0.5_Mo_0_, (**f**) Fe_0.5_Mo_0.05_, (**g**) Fe_0.5_Mo_0.1_, (**h**) Fe_0.5_Mo_0.15_.

The optical micrographs of the XZ planes of the as-printed alloys fabricated under the optimal processing parameters are presented in Figure 4a–g. All alloys exhibit surfaces free from large-scale porosity or unmelted particles. For the equiatomic FeCoCrNiMo_x_ alloy, when the Mo atomic fraction reaches 0.15, pronounced corrosion pits are observed along the sample edges. In contrast, for the non-equiatomic Fe_0.5_CoCrNiMo_x_ alloy, the Fe_0.5_Mo_0.15_ high-entropy alloy experiences severe edge cracking during the printing process. As shown in Figure 4h, macroscopic cracks are clearly visible within the as-printed specimen after sectioning along the build direction. Consequently, this composition was excluded from further investigation. In summary, for FeCoCrNi-based HEAs, printability progressively deteriorates with increasing Mo content.

**Figure 5:**
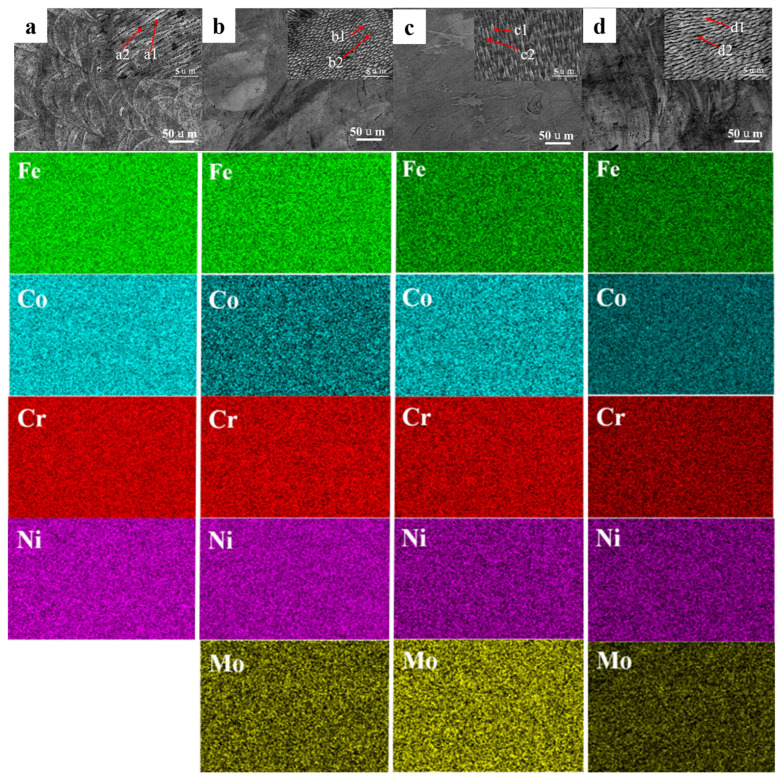

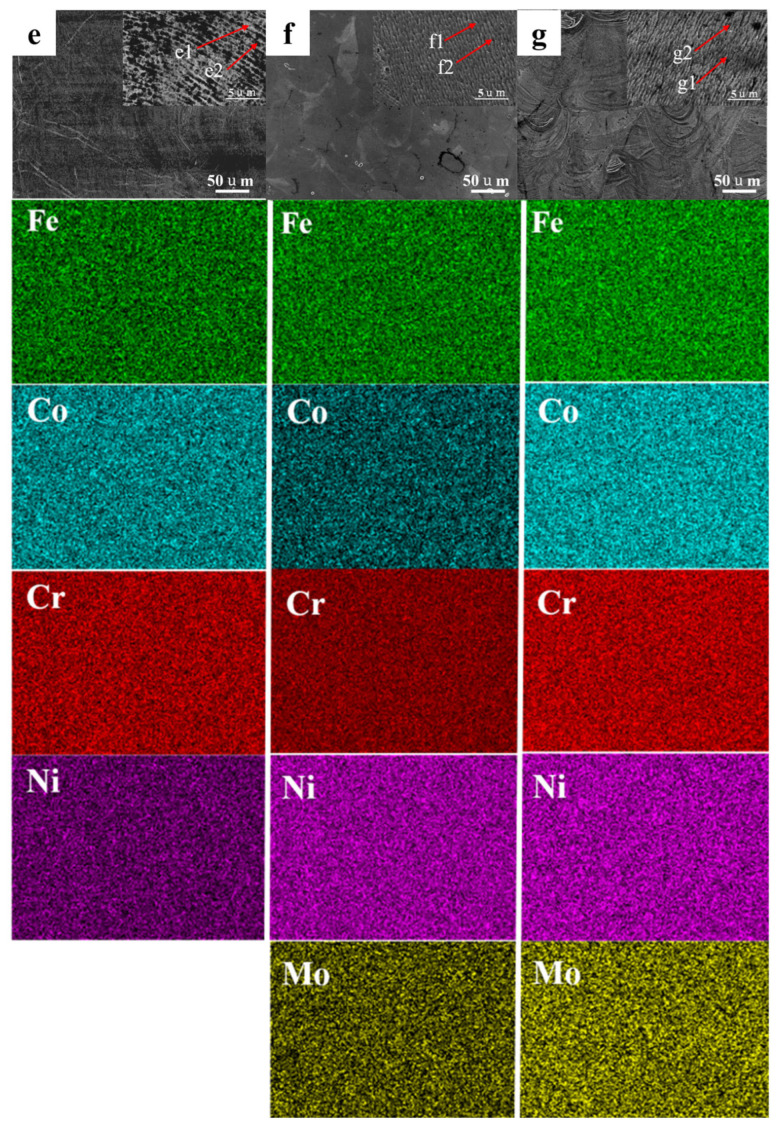
SEM images and EDS spectra of XZ plane of high-entropy alloys with various components in as-printed state (**a**) Mo_0_, (**b**) Mo_0.05_, (**c**) Mo_0.1_, (**d**) Mo_0.15_, (**e**) Fe_0.5_Mo_0_, (**f**) Fe_0.5_Mo_0.05_, (**g**) Fe_0.5_Mo_0.1_.

The SEM morphology in Figure 5 reveals that the samples prepared by L-PBF exhibit typical fish-scale-like interlocked stacked melt pools after corrosion. The melt pools are relatively regular in shape, with tightly overlapped boundaries between adjacent pools (as shown in Figure 5a–g). Upon magnification of individual melt pools, it is clear that the pools consist of numerous fine, elongated cellular substructures. The formation of these cellular structures is primarily attributed due to the ultrafast cooling conditions generated in the L-PBF process. Under rapid solidification conditions, the cellular substructures preferentially evolve in the direction of the highest temperature gradient, perpendicular to the melt pool boundary. (higher temperature gradient, G) towards the melt pool surface (lower G) [40,41]. Therefore, on a three-dimensional scale, these cellular substructures appear as columns in the solidification direction, while in the direction perpendicular to the solidification axis, they exhibit a cellular arrangement.

Furthermore, during the L-PBF process, if the input energy is relatively high, the molten layer not only affects the current layer but also significantly influences the previously solidified layer beneath, resulting in local remelting or heat treatment effects. Under this influence, the original dendritic structure continues to grow along its original orientation during the repeated heating and cooling cycles. This phenomenon facilitates the breaking of the grain structure’s single-layer boundary, forming columnar crystals that extend through multiple layers.

High magnification SEM analysis further reveals that the internal structure of each melt pool primarily consists of fine cellular substructures, which are highly dense and evenly distributed. Combined with the EDS point scan analysis results shown in Table 2, it is evident that the Fe, Co, and Ni elements in the FeCoCrNiMo_x_ and Fe_0.5_CoCrNiMo_x_ high-entropy alloys prepared by L-PBF are uniformly distributed, whereas the Cr and Mo elements exhibit compositional deviations within the cellular structures and along their boundaries. This suggests that Cr and Mo tend to accumulate at specific locations during rapid solidification and interface migration processes.

#### 3.1.4. Hardness Testing

**Figure 6:**
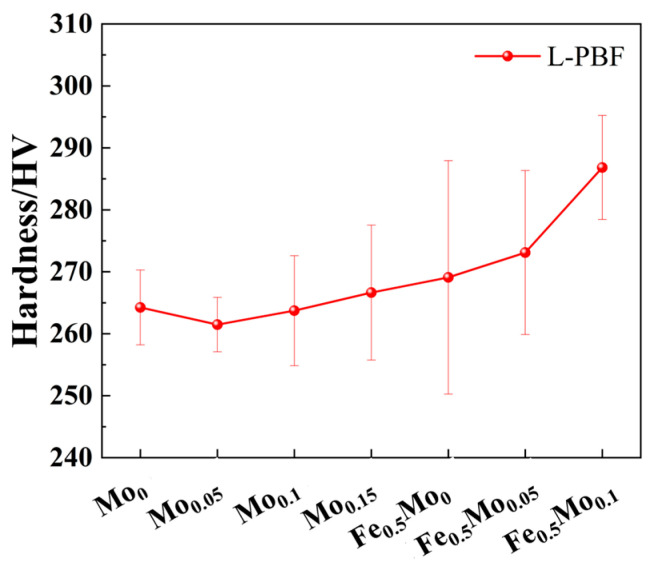
Hardness variation curves of HEAs with varying Mo contents fabricated via L-PBF.

Figure 6 illustrates the relationship between microhardness and Mo content for FeCoCrNiMo_x_ and Fe_0.5_CoCrNiMo_x_ HEAs. The results indicate that the overall microhardness of the CoCrNiFe_y_Mo_x_ does not vary significantly with increasing Mo content. For equiatomic FeCoCrNi-based HEAs, the hardness fluctuates slightly around 266.6 HV as the Mo atomic fraction increases. In contrast, the non-equiatomic Fe_0.5_CoCrNi-based HEAs exhibit a clear upward trend in Vickers hardness with increasing Mo content, Among them, Fe_0.5_CoCrNiMo_0.1_ shows the highest hardness, with an average hardness value of 286.82 HV.

### 3.2. Electrochemical Testing and Corrosion Morphology Analysis

#### 3.2.1. Electrochemical Testing

**Figure 7:**
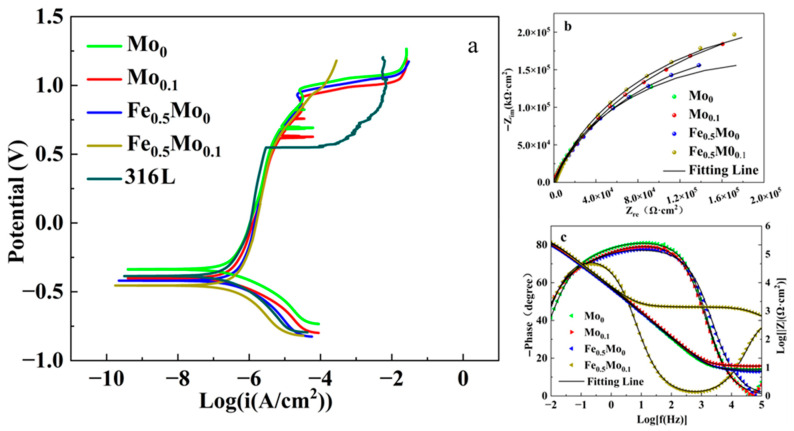
Electrochemical tests of HEAs with different Mo contents in a 3.5 wt.% NaCl solution at 25 °C (**a**) Tafel curve, (**b**) Nyquist diagram, (**c**) Bode diagram.

**Figure 8:**
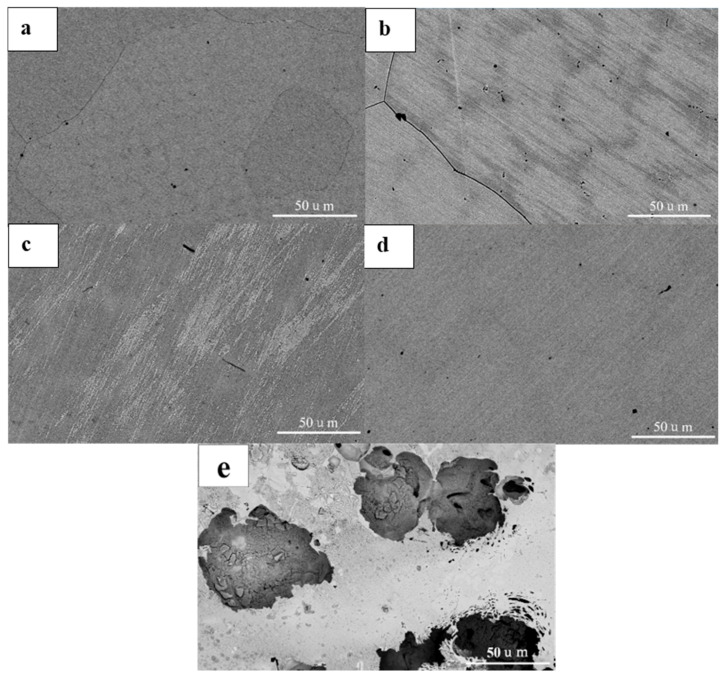
The corrosion morphology of CoCrNiFe_y_Mo_x_ HEAs after electrochemical tests in a 3.5 wt.% NaCl solution at 25 °C (**a**) Mo_0_, (**b**) Mo_0.1_, (**c**) Fe_0.5_Mo_0_, (**d**) Fe_0.5_Mo_0.1_, (**e**) 316L.

Electrochemical measurements were initially conducted at room temperature in a 3.5 wt.% NaCl solution for HEAs of various compositions as well as for 316L stainless steel. Electrochemical tests were first conducted in 3.5 wt.% NaCl solution at room temperature for HEAs with different compositions. The test results are shown in Figure 7. Analysis of the polarization curves of the four alloys revealed pronounced passivation plateaus, indicating the formation of protective oxide films. However, no significant differences were observed in the breakdown potentials, and no distinct pitting potential transitions appeared during anodic scanning. Together with the corroded morphologies shown in Figure 8, these results suggest that the passive films formed on CoCrNiFe_y_Mo_x_ and HEAs at 25 °C in 3.5 wt.% NaCl solution possess strong stability and resistance to pitting, remaining intact against Cl^−^ attack, and thus no pitting occurred. For 316L stainless steel, a distinct pitting potential inflection point was observed, and its passive region was markedly narrower than that of the FeCoCrNiMo high-entropy alloy. In addition, pronounced corrosion pits were found on the surface morphology after electrochemical testing, indicating that the corrosion resistance of 316L stainless steel is significantly inferior to that of the FeCoCrNiMo high-entropy alloy.

In order to further study the pitting corrosion behavior of CoCrNiFe_y_Mo_x_. additional electrochemical tests were carried out in 3.5 wt.% NaCl solution at 60 °C. This approach enabled characterization of the influence of Mo content on the pitting resistance of the alloys.

**Figure 9:**
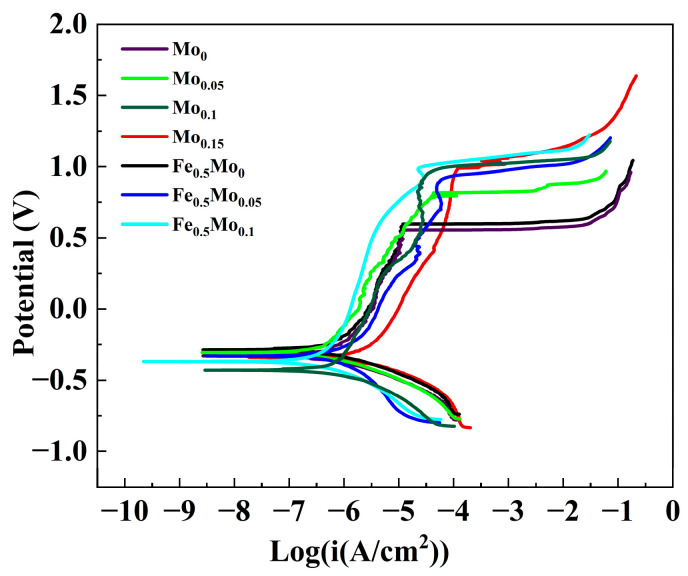
The polarization curves of CoCrNiFe_y_Mo_x_ HEAs in a 60 °C, 3.5 wt.% NaCl solution.

Figure 9 presents the potentiodynamic polarization curves of as-printed CoCrNiFe_y_Mo_x_ HEAs measured in NaCl solution at 60 °C. From these curves, the corrosion potential (E_corr_), corrosion current density (I_corr_), and pitting potential (E_pit_) were determined and are summarized in Table 3. The corrosion potential reflects the tendency of the alloy to undergo corrosion, the corrosion current density indicates the corrosion rate once corrosion occurs, and the pitting potential represents the alloy’s resistance to pitting corrosion.

**Figure 10:**
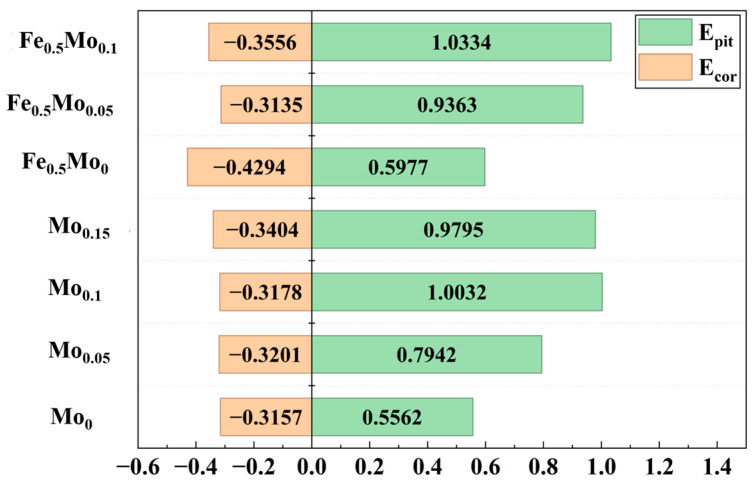
Electrochemical parameters of HEAs with different Mo contents.

As shown in Figure 9, HEAs with different Mo contents exhibit typical passivation behavior in NaCl solution. The values of E_corr_ and I_corr_, calculated using the Tafel extrapolation method, are listed in Table 3. The variation trends of E_pit_ and E_corr_ with Mo content are illustrated in Figure 10. With increasing solution temperature, the passive region of FeCoCrNi and Fe_0.5_CoCrNi high-entropy alloys is significantly reduced. The rates of anodic dissolution and cathodic reduction reactions at the electrode interface are markedly accelerated, while the diffusion rates of cations and oxygen anions through the film are also enhanced, which readily leads to compositional inhomogeneity of the film and the formation of voids or defects. In addition, in chloride-containing media, elevated temperature strengthens the penetration and competitive adsorption ability of Cl^−^ ions, thereby reducing corrosion resistance [42,43]. Consequently, in the corrosive medium at 60 °C, the passive region of Mo_0_ and Fe_0.5_Mo_0_ high-entropy alloys is notably diminished. Upon the addition of Mo, however, the pitting potential increases with Mo addition up to x = 0.1 and then decreases, with Mo_0.1_ displaying the highest E_pit_ of 1.003 V_SCE_. In the case of Fe_0.5_CoCrNi-based HEAs, Fe_0.5_Mo_0.1_ exhibits the maximum pitting potential of 1.034 V_SCE_. These results clearly indicate that the addition of Mo effectively enhances the resistance of FeCoCrNi-based HEAs to pitting corrosion in chloride-containing environments.

When comparing equiatomic FeCoCrNiMo_x_ alloys with the non-equiatomic Fe_0.5_CoCrNiMo_x_ counterparts, it is evident that, at the same Mo content, the non-equiatomic alloys exhibit superior pitting resistance. This improvement is attributed to their higher Cr and Mo concentrations, which strengthen the protective characteristics of the passive film and enhance resistance against chloride-induced localized corrosion.

**Figure 11:**
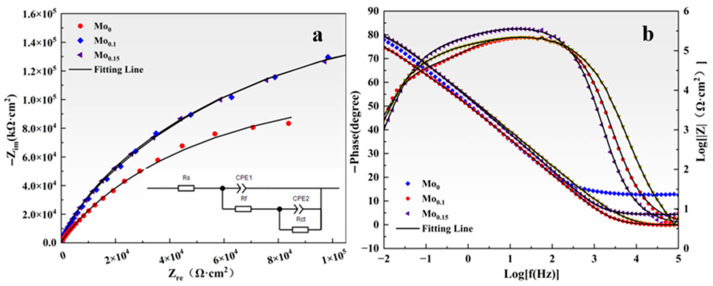
EIS curves of CoCrNiFe_y_Mo_x_ HEAs in a 3.5wt.% NaCl solution: (**a**) Nyquist diagram, (**b**) Bode diagram. The illustration in Figure a depicts a fitted circuit.

Electrochemical impedance spectroscopy was additionally used to assess the corrosion characteristics of Mo_0_, Mo_0.1_, and Mo_0.15_ alloys in NaCl solution. As shown in Figure 11a, all Nyquist plots exhibit similar semicircular impedance arcs, indicating that the corrosion behavior of FeCoCrNi-based alloys with varying Mo contents is broadly comparable. According to the principle of Nyquist analysis, the arc radius is indicative of the resistance value of the CoCrNiFe_y_Mo_x_ passive film: a larger radius corresponds to higher impedance of the passive film and thus better corrosion resistance [27]. It is evident from the figure that the semicircle radius first increases and then decreases with Mo addition, reaching its maximum when x = 0.1. This demonstrates that the Mo_0.1_ alloy possesses the most favorable corrosion resistance.

Figure 11b displays the Bode plots of alloys with different Mo contents. The Mo_0.1_ alloy shows the highest impedance modulus, approaching −80° over a broad frequency range, which suggests superior chemical stability and enhanced corrosion resistance. To further interpret the EIS results, the data were fitted using an equivalent circuit with time constants, as illustrated in the inset of Figure 11a [20,44,45]. Due to the non-uniformity of the electrode surface, a constant phase element (CPE) was introduced to simulate the non-ideal capacitive response.

The parameters obtained from fitting the equivalent circuit, including solution resistance (R_s_), charge transfer resistance (R_ct_), and constant phase element (CPE), are presented in Table 4. As the content of the Mo element increases, the R_ct_ value initially increases and then decreases, reaching its maximum for the Mo_0.1_ alloy. This indicates that charge transfer is most hindered in Mo_0.1_, which corresponds to the slowest corrosion rate. Since R_ct_ is inversely related to the corrosion rate, this confirms that the passive film on Mo_0.1_ is the most compact and protective. The exponent *n*, which describes the surface heterogeneity in the CPE, ranges from 0 to 1; *n* = 1 represents an ideal capacitor, while *n* = 0 corresponds to a pure resistor.

#### 3.2.2. Corrosion Morphology Analysis

**Figure 12:**
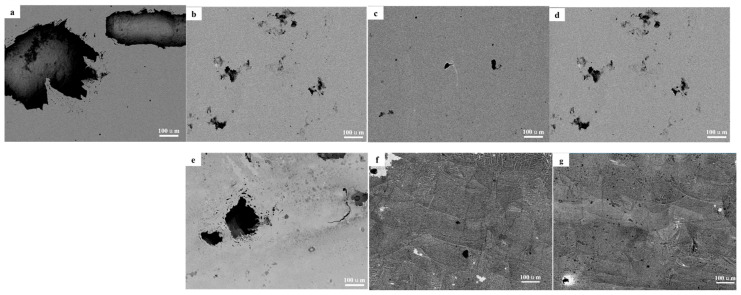
The surface morphology after corrosion (**a**) Mo_0_, (**b**) Mo_0.05_, (**c**) Mo_0.1_, (**d**) Mo_0.15_, (**e**) Fe_0.5_Mo_0_, (**f**) Fe_0.5_Mo_0.05_, (**g**) Fe_0.5_Mo_0.1_.

The corroded morphologies are presented in Figure 12. Corrosion pits of various sizes were observed, indicating that HEAs with different Mo contents all experienced corrosion to different extents. Severe corrosion occurred on the surfaces of Mo_0_ and Fe_0.5_Mo_0_ alloys, where large corrosion pits were accompanied by smaller pitting sites. This suggests that localized corrosion originated from pitting. Under the testing condition of 60 °C in 3.5 wt.% NaCl solution, the passive film on these alloys failed to provide sufficient protection, ultimately leading to the formation of large corrosion pits. By contrast, only slight pitting was detected on Mo_0.1_ and Fe_0.5_Mo_0.1_ alloys, demonstrating that the addition of even a small amount of Mo significantly improved the protective performance of the passive film.

For single-phase alloys, the passive film can be considered nearly continuous and homogeneous, and thus their corrosion resistance is mainly governed by the composition and structure of the passive film [22]. Therefore, to further elucidate the influence of Mo on the composition and structural characteristics of the passive film, XPS analysis was conducted on the passivation films formed on Mo_0_ and Mo_0.1_ alloys in the corrosive solution.

### 3.3. XPS Analysis of the Passive Film

**Figure 13:**
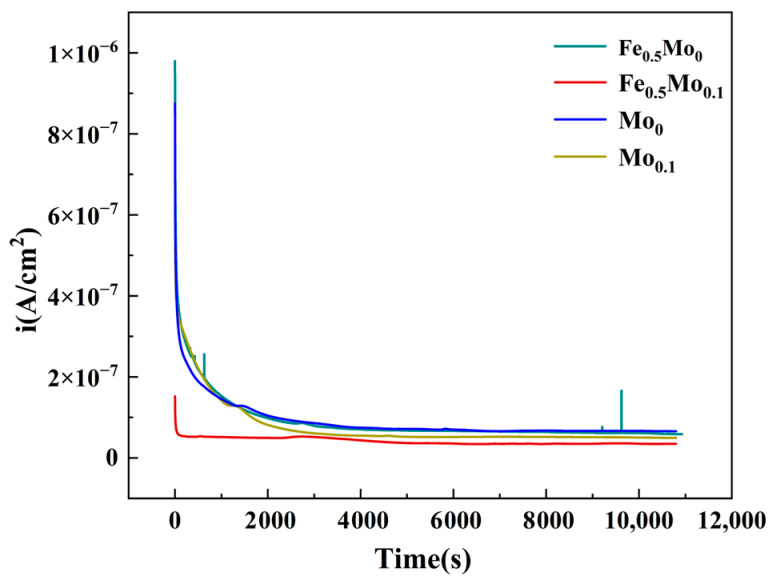
Constant potential polarization curves of alloys with different Mo contents in a corrosive medium at 0 V_SCE_ for 3 h.

Based on the electrochemical test results mentioned above, the addition of Mo can significantly enhance the corrosion resistance of high-entropy alloys (HEAs). The corrosion resistance of an alloy largely depends on the composition and structure of its passive film [31]. Therefore, XPS was employed to analyze and compare the passive films formed on the alloys. In this study, Mo_0_ and Mo_0.1_ alloys were polarized at a constant potential of 0 V_SCE_ for 3 h in a corrosive medium to form passive films. The evolution of current during the polarization process is shown in Figure 13. As the polarization progressed, the current gradually stabilized, indicating the formation of a stable passive film on the alloy surface. Among them, Fe_0.5_Mo_0.1_ exhibited the lowest passivation current density, suggesting that its passive film is the most compact and stable.

**Figure 14:**
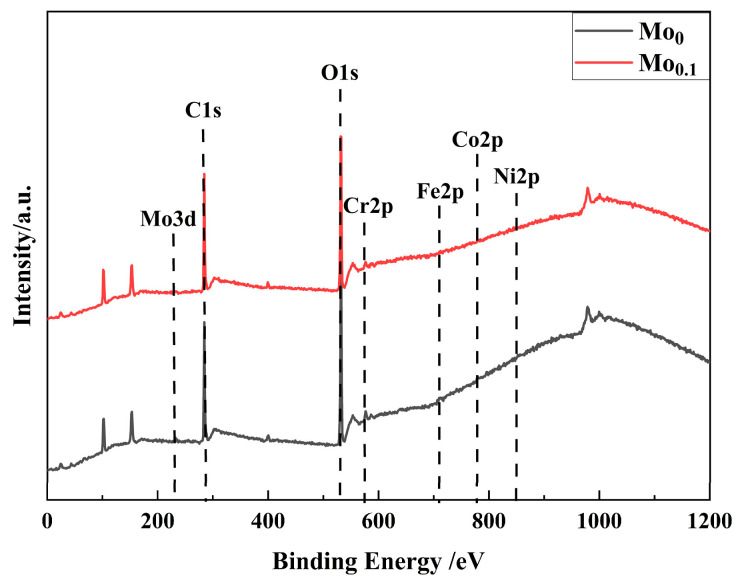
XPS full spectrum of the passivation films of Mo_0_ and Mo_0.1_ alloys.

**Figure 15:**
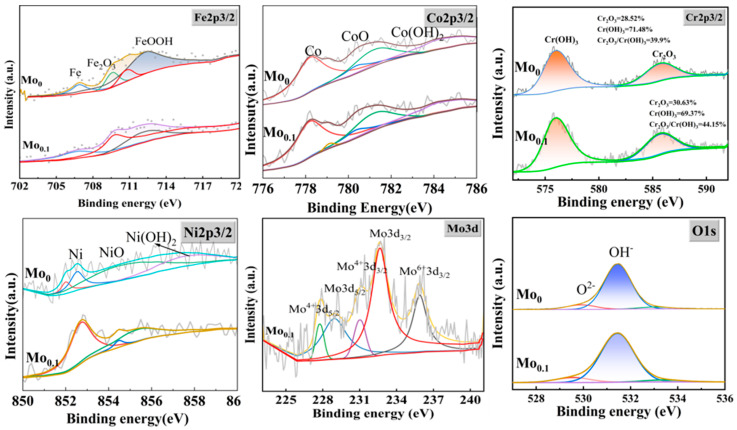
XPS fine spectra of the surface passivation films of Mo_0_ and Mo_0.1_ alloys in 3.5wt.% NaCl solution.

Figure 14 presents the full XPS spectra of the passive films on Mo_0_ and Mo_0.1_ alloys, showing the valence states of each element in the passive films. Figure 15 provides the fitting results for Fe 2p3/2 [26,46,47,48], Co 2p3/2 [26,49], Cr 2p3/2 [50,51,52], Ni 2p3/2 [26,53], Mo 3d [23,36,48], and O 1s [26,46] peaks in the passive films of Mo_0_ and Mo_0.1_ alloys. From the spectra, it can be seen that the Fe 2p3/2 peak in both alloys is divided into contributions from metallic Fe, Fe^3+^ in Fe(OH)O, and Fe^3+^ in Fe_2_O_3_. The Co 2p3/2 peak is split into three peaks corresponding to metallic Co, Co^2+^ in CoO, and Co^2+^ in Co(OH)_2_. The Cr 2p3/2 peak is divided into two components: Cr(OH)_3_ and Cr_2_O_3_. The Ni 2p3/2 peak is composed of three components corresponding to metallic Ni, NiO, and Ni(OH)_2_. The O1s peak is composed of two components corresponding to O^2−^ and OH^−^, indicating that the passive films on both alloys are mainly composed of oxides and hydroxides of the alloying elements. In Mo_0.1_, the ratio of Cr_2_O_3_ to Cr(OH)_3_ is 44.15%, which is higher than the 39.9% observed in Mo_0_. This suggests that the addition of Mo promotes the formation of more Cr_2_O_3_ in the passive film [54]. The excellent compactness and stability of Cr_2_O_3_ play a key role in enhancing the alloy’s corrosion resistance [55]. Therefore, the incorporation of a small amount of Mo can significantly improve the protective capacity of the alloy’s passive film in chloride-containing solutions. Additionally, the Mo3d peak in Mo_0.1_ reveals a more complex pattern. According to the literature [48], Mo^6+^ predominantly exists as Mo_4_^2−^ in the passive film, contributing to the inhibition of Cl^−^ ion-induced corrosion.

## 4. Conclusions

FeCoCrNiMo_x_ and Fe_0.5_CoCrNiMo_x_ high-entropy alloys were systematically investigated using SEM, EDS, XPS, and electrochemical measurements. The post-corrosion morphologies were also examined to elucidate their corrosion behavior in 3.5 wt.% NaCl solution at room temperature and 60 °C. The main conclusions are as follows:

1. All FeCoCrNiMo_x_ and Fe_0.5_CoCrNiMo_x_ alloys (x = 0, 0.05, 0.1, 0.15) exhibited an FCC crystal structure. Mo addition led to a pronounced increase in hardness, particularly in Fe_0.5_CoCrNiMo_x_ alloys, with Fe_0.5_CoCrNiMo_0.1_ achieving the highest hardness.
2. All alloys fabricated via L-PBF exhibited good corrosion resistance at room temperature. At 60 °C, Mo addition significantly improved pitting resistance. Under the condition of the same atomic ratio of Mo, the non-equiatomic Fe_0.5_CoCrNi alloys demonstrated superior corrosion resistance compared with equiatomic FeCoCrNi alloys.
3. XPS analysis of passive films formed under potentiostatic polarization revealed that Mo_0.1_ alloys contained a higher fraction of dense Cr_2_O_3_, which accounts for the enhanced pitting resistance of Mo-containing alloys.

## Figures and Tables

**Table 1:** The theoretical compositions of the designed CoCrNiFe_y_Mo_x_ alloys (wt.%).

Alloys	Fe	Co	Cr	Ni	Mo
Mo_0_	24.77	26.14	23.06	26.03	0
Mo_0.05_	24.25	25.59	22.58	25.49	2.08
Mo_0.1_	23.76	25.07	22.12	24.97	4.08
Mo_0.15_	23.28	24.57	21.68	24.47	6
Fe_0.5_Mo_0_	14.14	29.83	26.32	29.71	0
Fe_0.5_Mo_0.05_	13.8	29.12	25.7	29.01	2.37
Fe_0.5_Mo_0.1_	13.48	28.45	25.1	28.34	4.63
Fe_0.5_Mo_0.15_	13.17	27.81	24.54	27.69	6.79

**Table 2:** EDS analysis results of high-entropy alloys with various components (wt.%).

Alloys		Fe	Co	Cr	Ni	Mo
Mo_0_	a1	28	25.5	21.2	25.2	/
	a2	28.1	25.5	22.8	23.6	/
Mo_0.05_	b1	24.7	26.2	22.1	23.7	3.3
	b2	24.7	25.6	23.4	23.8	2.5
Mo_0.1_	c1	23.5	24.5	20.5	25.7	5.8
	c2	24	26.7	19.1	25.7	4.5
Mo_0.15_	d1	22.6	24.3	23.5	23.8	5.8
	d2	22.9	24.9	23.6	23.3	5.4
Fe_0.5_Mo_0_	e1	15	30.2	26.5	28.2	/
	e2	15.4	30.7	25.9	27.9	/
Fe_0.5_Mo_0.05_	f1	16.9	29.6	21.9	28.3	3.3
	f2	16.8	29.9	21.5	29	2.8
Fe_0.5_Mo_0.1_	g1	17.6	30.4	21.6	25.6	4.8
	g2	18	30.9	21	26	4.1

**Table 3:** Electrochemical Parameters of Various High Entropy Alloys.

Alloys	Ecorr (V_SCE_)	Icorr (A/cm^2^)	Epit (V_SCE_)
Mo_0_	−0.3157	1.371 × 10^6^	0.5562
Mo_0.05_	−0.3201	1.277 × 10^6^	0.7942
Mo_0.1_	−0.3178	1.294 × 10^6^	1.0032
Mo_0.15_	−0.3404	2.1267 × 10^6^	0.9795
Fe_0.5_Mo_0_	−0.4294	2.541 × 10^6^	0.5977
Fe_0.5_Mo_0.05_	−0.3135	6.173 × 10^6^	0.9363
Fe_0.5_Mo_0.1_	−0.3556	9.012 × 10^7^	1.0334

**Table 4:** Fitting parameters of equivalent circuits for impedance spectra of CoCrNiFe_y_Mo_x_ HEAs in a 60 °C, 3.5 wt.% NaCl solution.

Alloys	R_s_ (Ω·cm^2^)	CPE1 Y_0_(Ω^−1^·cm^−2^s^−n^)	n1	R_f_ (Ω·cm^2^)	CPE2 Y_0_(Ω^−1^·cm^−2^s^−n^)	n2	R_ct_ (Ω·cm^2^)
Mo_0_	3.92	4.34 × 10^−5^	0.893	24363	2.27 × 10^−5^	0.693	2.45 × 10^5^
Mo_0.1_	9.36	4.12 × 10^−5^	0.836	48225	1.39 × 10^−5^	0.635	3.48 × 10^5^
Mo_0.15_	7.24	2.61 × 10^−5^	0.933	39569	7.75 × 10^−6^	0.668	3.10 × 10^5^

## Data Availability

The original contributions presented in this study are included in the article. Further inquiries can be directed to the corresponding authors.

## Abbreviations

The following abbreviations are used in this manuscript:

HEA	High Entropy Alloy
FCC	Face-Centered Cubic
L-PBF	Laser powder bed fusion
SLM	Selective laser melting
IHT	Intrinsic heat treatment
SEM	Scanning electron microscope
EDS	Energy Dispersive Spectroscopy
EIS	Electrochemical impedance spectroscopy
OCP	Open-circuit potential
XPS	X-ray Photoelectron Spectroscopy
XRD	X-ray Diffraction
